# Surface‐enhanced Raman spectral biomarkers correlate with Ankle Brachial Index and characterize leg muscle biochemical composition of patients with peripheral arterial disease

**DOI:** 10.14814/phy2.12148

**Published:** 2014-09-17

**Authors:** Kim Cluff, Abby M. Kelly, Panagiotis Koutakis, Xiang N. He, Xi Huang, Yong Feng Lu, Iraklis I. Pipinos, George P. Casale, Jeyamkondan Subbiah

**Affiliations:** 1 Bioengineering, Wichita State University, Wichita, Kansas; 2 Biological Systems Engineering, University of Nebraska, Lincoln, Nebraska; 3 Division of General Surgery, Department of Surgery, University of Nebraska Medical Center, Omaha, Nebraska; 4 Department of Electrical Engineering, University of Nebraska, Lincoln, Nebraska; 5 Department of Surgery and VA Research Service, VA Nebraska‐Western Iowa Health Care System, Omaha, Nebraska; 6 Food Science and Technology, University of Nebraska, Lincoln, Nebraska

**Keywords:** Linear discriminant analysis, muscle biochemistry, partial least squares regression, peripheral arterial disease, Raman spectroscopy

## Abstract

Peripheral arterial disease (PAD) is characterized by atherosclerotic blockages of the arteries supplying the lower extremities, which cause a progressive accumulation of ischemic injury to the skeletal muscles of the lower limbs. This injury includes altered metabolic processes, damaged organelles, and compromised bioenergetics in the affected muscles. The objective of this study was to explore the association of Raman spectral signatures of muscle biochemistry with the severity of atherosclerosis in the legs as determined by the Ankle Brachial Index (ABI) and clinical presentation. We collected muscle biopsies from the gastrocnemius (calf muscle) of five patients with clinically diagnosed claudication, five patients with clinically diagnosed critical limb ischemia (CLI), and five control patients who did not have PAD. A partial least squares regression (PLSR) model was able to predict patient ABI with a correlation coefficient of 0.99 during training and a correlation coefficient of 0.85 using a full cross‐validation. When using the first three PLS factor scores in combination with linear discriminant analysis, the discriminant model was able to correctly classify the control, claudicating, and CLI patients with 100% accuracy, using a full cross‐validation procedure. Raman spectroscopy is capable of detecting and measuring unique biochemical signatures of skeletal muscle. These signatures can discriminate control muscles from PAD muscles and correlate with the ABI and clinical presentation of the PAD patient. Raman spectroscopy provides novel spectral biomarkers that may complement existing methods for diagnosis and monitoring treatment of PAD patients.

## Abstract (precis)

Raman spectroscopy is capable of detecting and measuring unique biochemical signatures of skeletal muscle. These signatures can discriminate control muscles from peripheral arterial disease (PAD) muscles and correlate with the ABI and clinical presentation of the PAD patient. Raman spectroscopy provides novel spectral biomarkers that may complement existing methods for diagnosis and monitoring treatment of PAD patients.

## Introduction

Peripheral arterial disease (PAD), characterized by atherosclerotic blockages of the arteries supplying the lower extremities in association with muscle damage and limb dysfunction, produces a considerable public health burden affecting 12–20% of Americans age 65 and older (Mahoney et al. 2008, 2010; Roger et al. 2011). PAD decreases the blood flow to the affected legs, causing an accumulation of ischemic injury to the leg that is, reflected in altered metabolic processes (mitochondrial dysfunction and oxidative damage) and gradual worsening of the histology of affected skeletal muscles (myofiber degeneration and fibrosis), a condition identified as PAD myopathy (Hedberg et al. 1988; Pipinos et al. 2008b; Cluff et al. 2013; Weiss et al. 2013). The principal clinical manifestations of PAD are claudication (exercise‐induced leg pain and dysfunction) and in severe cases critical limb ischemia (CLI) which includes foot pain at rest (ischemic rest pain) and tissue loss (nonhealing ulcers and gangrene). Ankle Brachial Index (ABI, a measure of limb hemodynamics) is the standard, noninvasive method for diagnosing presence and severity of atherosclerotic blockages in arteries supplying the legs and for monitoring the hemodynamic effects of treatment interventions for PAD. ABIs are measured as the ratio of the systolic pressure in the ankle to that in the arm. Stenoses and occlusions of the arteries supplying the legs lower the ankle blood pressure and yield a low ABI, which is the hemodynamic hallmark of PAD. PAD is diagnosed by resting ABI values of <0.90, with values of 0.90–1.2 considered normal. Other more detailed methods of arterial assessment, such as ultrasonography and angiography (based on computerized tomography, magnetic resonance, or standard X‐Ray‐based evaluation), also are used by clinicians to evaluate patients with PAD. All of these methods, like ABI, focus on the diseased arterial tree and their main limitation is that they do not evaluate the effects of PAD on the chronically ischemic end‐organ which is the leg and its muscles.

Recently, ultrastructural and biochemical studies have sought to characterize the muscle damage (myopathy of PAD) occurring secondary to the vascular disease (Pipinos et al. 2008a,b,c; Cluff et al. 2013; Weiss et al. 2013). These studies have demonstrated that long‐term, leg ischemia as well as repeated cycles of ischemia followed by reperfusion, occurring with basic daily activities; induce the generation of reactive oxygen species (ROS) in the PAD muscle. ROS produce oxidative damage to the tissues of the ischemic limb, leading to degeneration and dysfunction of cells and their organelles (mitochondria) and activation of inflammatory pathways (McDermott et al. 2004; Pipinos et al. 2008a,c; Brevetti et al. 2010; Weiss et al. 2013). Work from our group and others have shown that precise determination of the degree and nature of damage in the ischemic muscles can be central to clinical decision making (Brass and Hiatt 2000; Brass et al. 2004; Pipinos et al. 2008a,c; Brevetti et al. 2010; Berger and Hiatt 2012; Hiatt et al. 2012) first because it can measure the severity of PAD at baseline, when the patient first presents to the physician, and second because it can help determine how effective and clinically relevant a therapeutic intervention for PAD is based on the effects of the intervention on the ischemic skeletal muscle.

Raman spectroscopy is a label‐free analytical tool that uses the scattering properties of light to characterize molecular vibrations within a tissue sample, yielding a molecular fingerprint. Raman spectroscopy provides information‐rich molecular profiles and has potentially important clinical applications for real‐time in situ evaluation of living tissues (Hanlon et al. 2000). Raman spectral analysis has produced distinct molecular profiles of diseased bronchial tissue, brain tissue, stomach, and breast tissue (Nijssen et al. 2002; Koljenovic et al. 2004; Bonifacia et al. 2010; Kirsch et al. 2010). Other investigations have demonstrated that Raman spectroscopy allows construction of chemical maps of subcellular structures such as, the nucleus, and mitochondria (Bonnier et al. 2010; Miljković et al. 2010). Applications of Raman spectroscopy have been further broadened by making use of metallic nanostructured surfaces that produce a multifold increase in the Raman scattering signals, referred to as surface‐enhanced Raman spectroscopy (SERS; Sharma et al. 2012).

The objective of this study was to correlate hemodynamic limitation (determined as ABI) and clinical presentation (claudication vs. CLI) of the PAD patient with alterations in the biochemical profile of their chronically ischemic leg muscle as determined by Raman spectroscopy. Our central hypothesis is that SERS signatures from human biopsy samples (gastrocnemius muscle) correlate with hemodynamic limitation (ABI) and disease stage in the legs of the evaluated subjects. The rationale that underlies this research is that SERS signatures may become valuable clinical biomarkers in the practice of vascular surgery. Such biomarkers can be useful complements to new and expanded diagnostic methods that can include (along with clinical and hemodynamic parameters) direct measurements of tissue damage. These diagnostic methods can help to stratify patients based on the degrees of damage present in their skeletal muscle. Furthermore, these biomarkers can be used as surrogate endpoints that can assess and predict the effectiveness of a therapeutic intervention.

## Materials and Methods

### Tissue samples

The tissue collection protocol was approved by the Institutional Review Board, and all subjects gave informed consent. We collected muscle biopsies from the gastrocnemius (calf muscle) of five patients with clinically diagnosed critical limb ischemia (CLI), five patients with clinically diagnosed claudication, and five control patients (resting ABI > 0.9) who did not have lower limb impairment. Demographic data, from these patients, including ABI's, and age are presented in Table 1. The biopsies were acquired with a Bergstrom needle, fixed in methacarn, and embedded in paraffin. Two 4 *μ*m cross sections, from each biopsy, were mounted on slides for Raman spectra acquisition. Prior to Raman spectral acquisition, the slide specimens were deparaffinized with xylene, rehydrated through a series of ethanol washes, and allowed to air dry.

**Table 1.** Demographics of patients with peripheral arterial disease and control patients

	Control	Claudication	CLI
Number of subjects	5	5	5
Mean age (years) ± SD	63.2 ± 6.02	67.8 ± 9.58	58.4 ± 2.82
Ankle Brachial Index (ABI)	1.06 ± 0.03	0.55 ± 0.03	0.19 ± 0.06

CLI, critical limb ischemia.

### Surface‐enhanced Raman spectroscopy (SERS)

All tissue specimens were mounted on nanostructured gold‐slides (AU.1000.ALSI; Platypus Technologies, Madison, WI) for SERS. Additionally, two control and two PAD tissue specimens were mounted on conventional glass slides for comparison of the nonenhanced Raman signal with the SERS signal. An inVia Raman microscope (Renishaw, Gloucestershire, UK) spectral imaging system was used to acquire Raman and SERS spectra of human muscle biopsy specimens. This system is a high‐sensitivity research grade system that supports multiple excitation lasers and allows acquisition of information‐rich spectral images.

Confocal Raman spectral signatures were collected within the finger print region (303–1901 cm^−1^) using a 514 nm excitation laser (with a spot size of ~20 *μ*m), with a 50× magnification objective. The tissue was photo‐bleached for 60 sec and then the spectrum was collected with a 10 sec exposure time. From each tissue specimen, approximately 10 spectra were collected from the center of 10 individual myofibers and then averaged (after data preprocessing) to represent the tissue specimen.

### Data preprocessing

**Figure 1.**
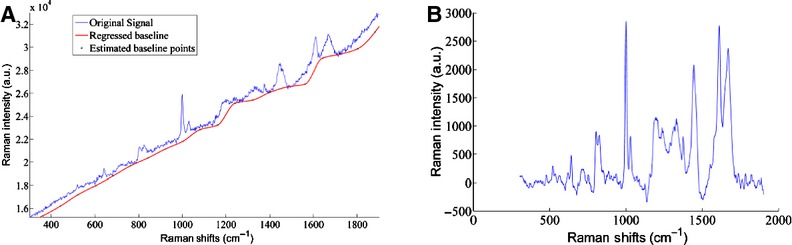
(A) Raman spectra were baseline corrected using a 5th order polynomial with the Vancouver Raman algorithm. (B) The extracted Raman signal after baseline correction.

Data preprocessing of the raw SERS spectra included baseline correction and normalization techniques. The Raman spectra were baseline corrected using the Vancouver Raman algorithm (Zhao et al. 2007) with a 5‐point boxcar smoothing and a 5th order polynomial fit, shown in Figure 1. The Vancouver Raman algorithm is a robust iteratively modified multi‐polynomial fitting algorithm that removes intrinsic autofluorescence background signals and improves signal‐to‐noise ratios (Lieber and Mahadevan‐Jansen 2003; Afseth et al. 2006; Zhao et al. 2007; Beier and Berger 2009). Following baseline correction, the spectra were normalized using the standard normal variate (SNV) technique. SNV normalization is a common mathematical transformation for spectral data and is designed to remove multiplicative error and preserve the linear relationship between the spectral signal and sample concentration (Barnes et al. 1989; Rinnan et al. 2009).

### Model development

A partial least squares regression (PLSR) model was developed using the entire spectral region from 303–1901 cm^−1^ to predict patient ABI. The PLSR algorithm combines techniques used in principal component analysis (PCA) and multiple linear regression and attempts to quantify the strength of the relationship between the response variable and a set of predictor variables (Geladi and Kowalski 1986). PLSR searches for principal components (called factors) that are orthogonal to each other and tries to relate them to the response variable. The variation present in the response variable can be summarized into a few PLS factors. The first PLS factors that explain the most variance in the data can then be used to build a regression model. An analysis of the *β*‐coefficients on the partial least squares regression model can identify which Raman shifts had the most weight in the predictive PLSR model. A full cross‐validation procedure (a common statistical analysis technique for estimating model performance) was performed on the data set to evaluate the performance of the predictive model. Cross‐validation is a standard multivariate statistical technique often used on small data sets to validate a model, assess stability, and determine how well it will perform on future data sets (Hastie et al. 2009). The cross‐validation technique rotates the membership of the samples (during training) to ensures that the results are not membership dependent (i.e., training group and validation group) and to ensure that the model is not overfitting the data.

In addition to the PLSR model, a discriminant model was developed as well using the PLS factor scores from the PLSR model to classify patients as control, claudicating, or CLI. The use of discriminant analysis in combination with PLS factor scores is a common multivariate statistical method to establish a mathematical rule that separates two or more classes from each other (Fisher 1936; Anderson 1984). Once the discriminant model has been derived, it can be used to classify new observations (Johnson and Wichern 2007). The discriminant model performance was evaluated with a full cross‐validation procedure as well.

## Results

### Surface‐enhanced Raman spectroscopy

**Figure 2.**
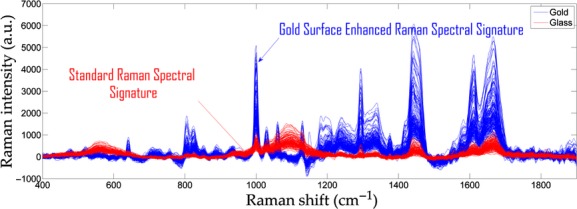
Surface‐enhanced Raman spectroscopy (SERS) enhances the spectral signature of myofibers mounted on nanostructured gold‐slides when compared to myofibers mounted on glass slides.

Figure 2 shows the difference between standard Raman spectroscopy (*n* = 143 myofibers mounted on glass slides from two control and two PAD tissue specimens) and SERS (*n* = 150 myofibers mounted on nanostructured gold‐slides from two control and two PAD tissue specimens). The Raman effect deals with the inelastic scattering of light, resulting in a scattered photon that has a shift in frequency from that of the excitation laser. In general, Raman scattering produces a very weak signal due to the fact that only a small fraction, approximately 1 in 10 million, of photons exhibit the Raman effect. SERS is a surface‐sensitive technique that can enhance the Raman signal by 10 fold (Sharma et al. 2012). From Figure 2, it is readily apparent that SERS enhances the spectral signature of myofibers mounted on nanostructured gold‐slides when compared to myofibers mounted on glass slides. The tissue mounted on the nanostructured gold‐slides produced sharper and more intense Raman peaks. Hence, all further analyses were performed based on the tissue specimens mounted on gold‐slides for SERS.

### Partial least squares regression and discriminant analysis

**Figure 3.**
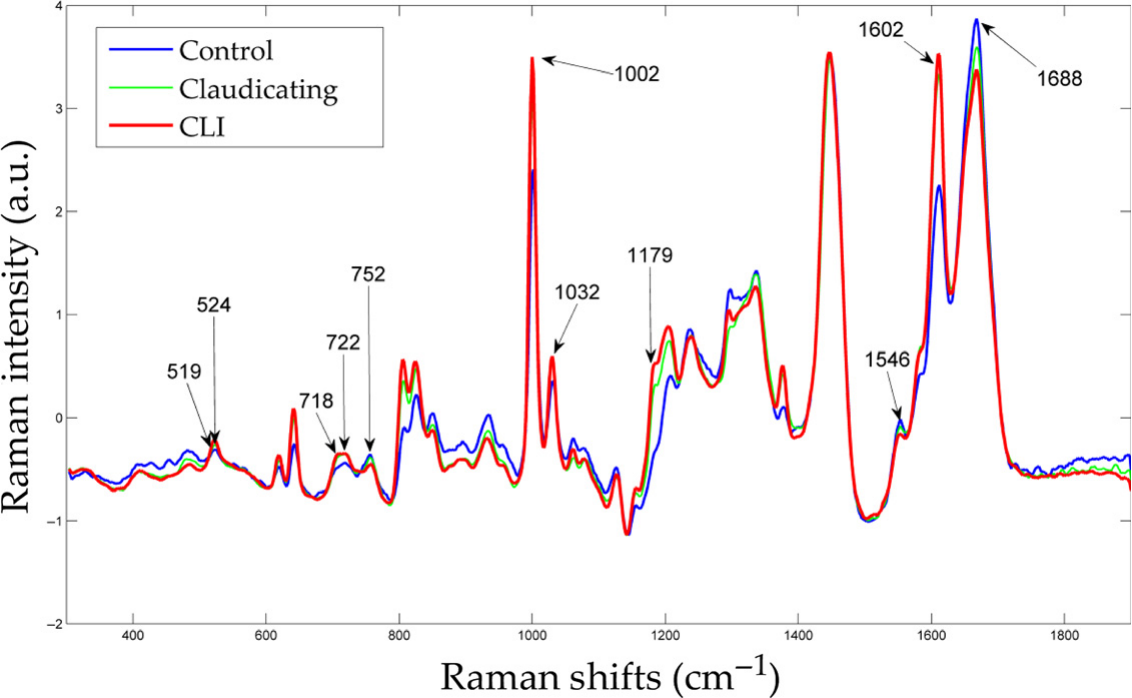
Average control (CON), claudicating (MOD), and critical limb ischemia (SEV) patient SERS.

**Figure 4.**
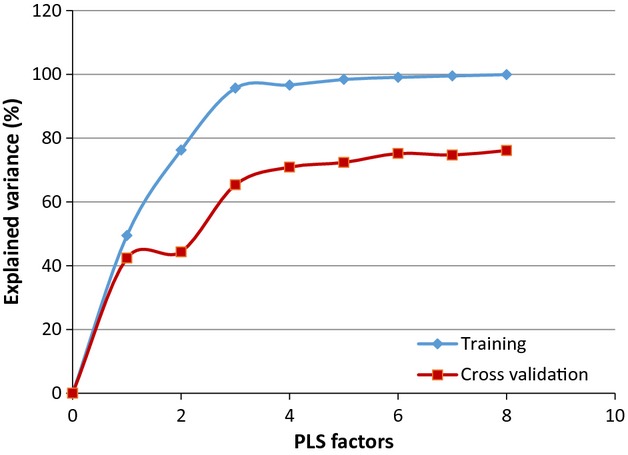
Eigenvalues of partial least squares factors. The first six PLS factors account for most of the variance (>75%).

**Figure 5.**
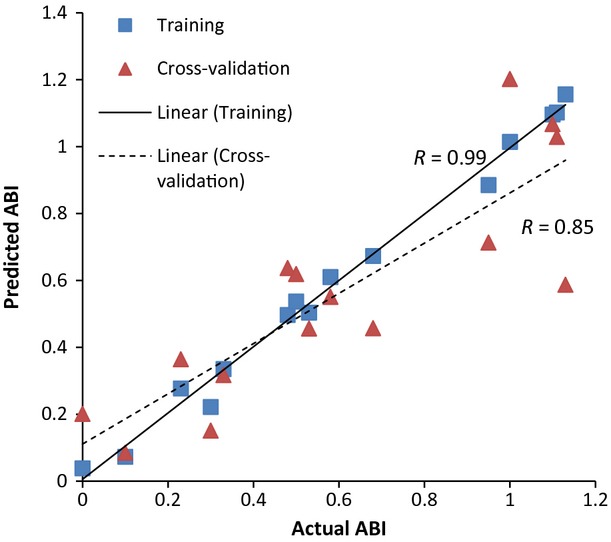
Partial least squares regression model using six PLS factors was able to predict patient ABIs with a correlation coefficient of 0.99 and 0.85 in training and full cross‐validation, respectively.

**Figure 6.**
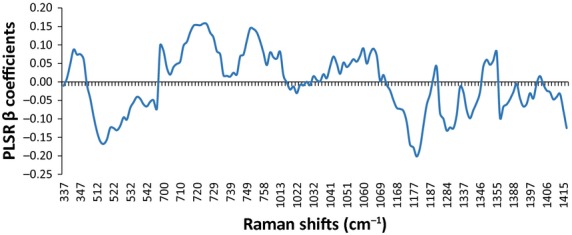
Partial least squares regression *β* coefficients.

Figure 3 presents the averaged baseline‐corrected SNV SERS for control, claudicating, and CLI patient specimens, which were used to develop the PLSR model. Nearly all of the variance (99%) in the response variable (patient ABI) was accounted for by the first six PLS factors, as shown in Figure 4. Using the first six PLS factors, the PLSR model was trained and the predictive performance was evaluated using a full cross‐validation procedure. The PLSR model was able to predict patient ABIs with a correlation coefficient of 0.99 during training and a correlation coefficient of 0.85 using a full cross‐validation, show in Figure 5. The *β*‐coefficients for the PLSR model are presented in Figure 6, which can be used to identify key wavenumbers that influence the PLSR model.

**Figure 7.**
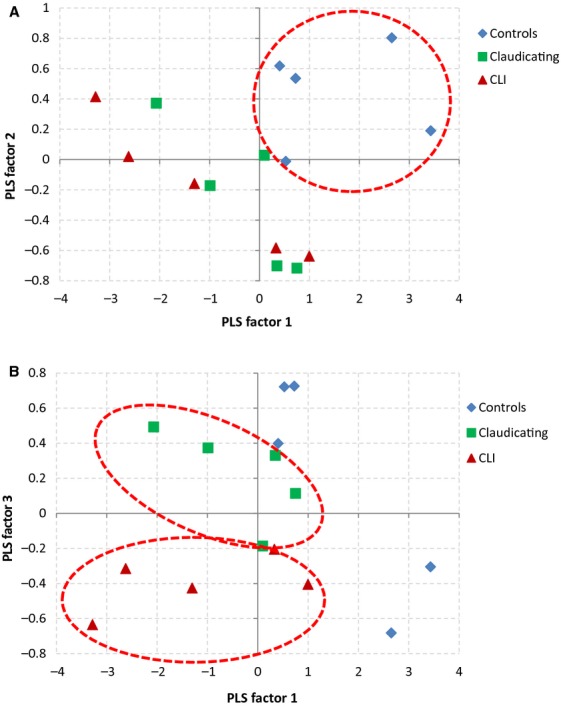
PLS factor scores plot. (A) PLS 1 and 2 grouped and separated control patients from claudicating and critical limb ischemia (CLI) patients. (B) PLS 1 and 3 separated claudicating and CLI patients. These three PLS factors in a discriminant analysis with cross‐validation, classified patients with 100% accuracy.

Further, a plot of the first three PLS factor scores (Fig. 7) displays clear separation of controls, claudicating, and CLI patients. When using the first three PLS factor scores in combination with linear discriminant analysis a discriminant model is able to correctly classify the control, claudicating, and CLI patients with 100% accuracy. Evaluating the discriminant model performance and stability using a cross‐validation procedure also yielded 100% accuracy in patient classification. Table 2 presents the classification table for the discriminant model cross‐validation results. The combination of PLS factors 1 and 2 (Fig. 7A) clearly group and separate control patients from claudicating and CLI patients, while, PLS factor 3 (Fig. 7B) clearly separates claudicating patients from CLI patients.

**Table 2.** Patient classification with a discriminant model and cross‐validation results

	Predicted membership				Accuracy %
	Control	Claudication	CLI	Total	
Actual membership					
Control	5	0	0	5	100
Claudication	0	5	0	5	100
CLI	0	0	5	5	100
Total	5	5	10	15	100

CLI, critical limb ischemia.

## Discussion

Raman spectroscopy is a powerful optical tool capable of detecting biochemical composition within biological tissue, without the use of labeling procedures. In this study, we have demonstrated that Raman spectroscopy is capable of predicting the ABIs and disease stage of PAD patients based on the biochemical fingerprint of their gastrocnemius. The PLSR model was able to correlate Raman spectral information with patient ABIs with a high level of correlation (*R*‐value = 0.85). Moreover, when combining PLS factor scores with discriminant analysis, the model is able to classify patients with 100% accuracy. To our knowledge, this is the first study to produce a Raman spectral profile of human PAD muscle. Further, very few studies, using other techniques, have been able to correlate patient ABIs with biochemical biomarkers in the serum or the ischemic limbs of PAD patients (Brevetti et al. 2006; Daskalopoulou et al. 2008; Anderson et al. 2009; Syvänen et al. 2011; Weiss et al. 2013). This study is novel because it uses Raman spectroscopy to produce a molecular profile of PAD muscle that accurately predicts patient ABI and clinical presentation in a manner that has not been done before.

An examination of the PLS regression *β*‐coefficients (Fig. 6) gives an indication of which wavenumbers had the most important impact on predicting patient ABIs. From Figure 6, it can be seen that wavenumbers 519, 524, 718–722, 752–755, and 1179 cm^−1^ had the largest *β* coefficients and thus indicate that these are key Raman spectral bands that predict the patient ABIs. The band occurring at 519 cm^−1^ was attributed to phosphatidylinositol, a phospholipid component of the cell membrane of eukaryotic cells (Lakshimi et al. 2002; Movasaghi et al. 2007). On average, this band had a higher intensity for CLI patients than control patients and a negative relationship with ABIs. The band appearing at 524 cm^−1^ was assigned to both the S‐S disulfide stretching in proteins and phosphatidylserine (Stone et al. 2004; Movasaghi et al. 2007). Phosphatidylserine is also a phospholipid component of the cell membrane that is, usually present on the inner leaflet of the bilayer, but becomes exposed at the cell surface during apoptosis (Verhoven et al. 1995). On average, this band had a higher intensity for CLI patients than controls and may indicate higher levels of apoptosis occurring in advanced stages of muscle degeneration (Mitchell et al. 2007). The 718 cm^−1^ band correlates with C‐N vibrational stretching in phosphatidylcholine lipid heads, which appear in ample amounts in mitochondrial membranes (Stone et al. 2002; Alberts et al. 2007; Movasaghi et al. 2007). This band had a higher intensity in PAD patients compared to control patients and may be indicative of higher mitochondrial content. Other studies of mitochondria physiology in PAD muscle also have reported increased mitochondrial content associated with mitochondrial dysfunction and compromised bioenergetics in PAD patients (Bylund et al. 1976; Lundgren et al. 1989; Pipinos et al. 2008c). However, it should be noted that phosphatidylcholine is not just present in mitochondria and appears in many other locations as well. Therefore, the increased intensity in this band also may indicate possible accumulations of debris or could be due to other lesions seen commonly in the ischemic myofibers, such as target lesions, which appear to represent damaged macromolecules and organelles that cannot be effectively processed and cleared from the injured myofibers. The 752 cm^−1^ band is attributed to porphyrin breathing mode, and is a direct measure of heme groups from hemoglobins providing an informative status of red blood cells (Deng et al. 2005; Movasaghi et al. 2007). This band may be reflecting the reduced blood flow in the lower limb musculature caused by stenosis of the arteries in PAD patients.

Other prominent bands occurring at 1546 cm^−1^ and 1688 cm^−1^ are attributed to the reduced form of nicotinamide adenine dinucleotide (NADH; Deng et al. 1989). The Raman scattering band occurring at 1032 cm^−1^ is attributed to the oxidized form of NAD^+^ (Yue et al. 1986). Based on the Raman spectra (Fig. 3), controls had higher intensities in NADH bands (1546 and 1688 cm^−1^) compared to PAD patients. This suggests that PAD patients have reduced levels of NADH and increased levels of NAD^+^. NADH is an essential coenzyme, within the mitochondria, and a key participant in the oxidative phosphorylation process. The NAD:NADH ratio plays an important role in the regulation of the intracellular redox state and is considered as an indicator of the metabolic state of the cell (Lin and Guarente 2003). Many metabolic pathways, especially the glycolytic and tricarboxylic acid pathways, have enzymes that are regulated by the NAD:NADH ratio (Belenky et al. 2007). Furthermore, cellular respiration studies indicate that increased production of ROS is linked to oxidation of NADH to NAD^+^ (Zuo and Clanton 2005; Sriramoju et al. 2008). The finding therefore, of an increased NAD:NADH ratio in PAD samples is likely reflective of a compromised metabolic and redox state in the PAD muscle (Pipinos et al. 2006; Makris et al. 2007; Weiss et al. 2013) and the particular band could be used as a Raman spectral biomarker of altered energy metabolism and oxidative stress in PAD limb musculature.

Raman scattering band observed at 1602 cm^−1^ has been associated with human mitochondria (Huang et al. 2004; Pully and Otto 2009). Figure 3, shows that PAD patients have a higher 1602 cm^−1^ band than control patients and suggests higher mitochondrial content in PAD patients. Again this is in agreement with other PAD studies that have demonstrated that PAD muscle has increased mitochondrial content (Brass and Hiatt 2000; Pipinos et al. 2008b). Although there is an increase in mitochondrial content in PAD muscle, respiration or oxidative phosphorylation is still lower when compared to the controls due to mitochondrial dysfunction (Makris et al. 2007; Pipinos et al. 2008b). Mitochondrial dysfunction can produce reactive oxygen species, which in turn could damage the phospholipid mitochondrial membrane system.

Although this study presents spectral biomarkers that are in agreement with other PAD studies, this study does have limitations. This study was performed using a small sample set of only 15 patients and its results should be further validated with a larger data set. As the severity of PAD progresses in the effected limb, there are likely to be intermediate states which may not have been captured in this study due to the sample size. These intermediate states could lead to misclassifications, thus decreasing the accuracy of the model, although the current results are promising. Likewise, only a cross‐validation procedure was implemented to assess the stability of the model as opposed to a true validation. A true validation on a new and larger data set will better ensure the accuracy of the model. Finally, the Raman spectral signal is a complicated mixed biochemical profile that can be difficult to interpret. Researchers have developed Raman spectral libraries of biological tissues to aid in the interpretation of Raman spectra (Movasaghi et al. 2007). However, the interpretation should be validated with controlled studies that vary concentration of the molecule and validate that the spectral peak changes accordingly. Therefore, further biochemical analysis should be performed to isolate and validate the presence of the suspected biochemical profile of PAD muscle tissue.

### Perspectives and significance

In this study, we have demonstrated that Raman spectroscopy is a powerful bioanalytical tool that identified biochemical‐based signatures unique to ischemic muscle. These signals predicted compromised hemodynamics in the legs of PAD patients and severity of clinical disease. Raman spectroscopy provides novel spectral biomarkers that may complement existing diagnostic and treatment monitoring methods for patients with PAD. Confirmation of the specificity and sensitivity of these spectral biomarkers may lead to novel techniques that can monitor PAD progression in the affected legs of patients by providing information that reflects the underlying pathophysiology and allows the development of individualized therapy. The long‐term goal of this research is the noninvasive, in vivo and in situ*,* quantification of progression or regression in PAD muscle degeneration as that would facilitate patient‐specific care and would have the greatest impact on disease management.

## Acknowledgments

The authors gratefully acknowledge Stanley Swanson and Karen Dulany from the University of Nebraska Medical Center for preparing tissue biopsy samples.

## Conflict of Interest

None declared.
